# Empirical dietary inflammatory index and lifestyle inflammation score relationship with obesity: A population‐based cross‐sectional study

**DOI:** 10.1002/fsn3.3660

**Published:** 2023-09-13

**Authors:** Niloufar Saber, Mahdieh Hosseinzadeh, Sakineh Shab‐Bidar, Masoud Mirzaei, Azadeh Najarzadeh, Seyedeh Tayebeh Rahideh

**Affiliations:** ^1^ Department of Nutrition, School of Public Health Iran University of Medical Sciences Tehran Iran; ^2^ Nutrition and Food Security Research Center, School of Public Health Shahid Sadoughi University of Medical Sciences Yazd Iran; ^3^ Department of Nutrition, School of Public Health Shahid Sadoughi University of Medical Sciences Yazd Iran; ^4^ Department of Community Nutrition, School of Nutritional Sciences and Dietetics Tehran University of Medical Sciences Tehran Iran; ^5^ Yazd Cardiovascular Research Center Shahid Sadoughi University of Medical Sciences Yazd Iran

**Keywords:** abdominal obesity, cross‐sectional study, empirical dietary inflammatory index, general obesity, lifestyle inflammatory score

## Abstract

The present study aimed to investigate the association between the empirical dietary inflammatory index (EDII) and lifestyle inflammatory score (LIS) with general and abdominal obesity in Iranian adults using data from the Yazd Health study (YaHS). This cross‐sectional study was conducted using the information of participants of the YaHS study. The dietary assessment was conducted using a validated food frequency questionnaire (FFQ) and anthropometric measurements assessed by standard protocols. The inflammatory potential of diet and lifestyle were calculated using EDII and LIS scores. We also created a combinational index of EDII and LIS as an EDII‐LIS score. General and abdominal obesity were defined based on body mass index (BMI), waist circumference (WC), and waist‐to‐hip ratio (WHR) cut points, respectively. The odds ratio (OR) and 95% confidence interval (CI) of general and abdominal obesity across tertiles of EDII and LIS were estimated using logistic regression analyses, adjusted for potential confounders. A significant association was found between a higher EDII score and general obesity (OR: 1.21, 95% CI: 1.04–1.41, *p* trend: .016), however, there was no significant association between EDII and both definitions of abdominal obesity. Participants in the highest versus lowest tertile of LIS had higher odds of increased abdominal obesity (OR_WC_: 37.0, 95% CI: 28.8–47.5, *p* trend <.001, OR_WHR_: 3.30, 95% CI: 2.65–4.11, *p* trend <.001). In addition, there was also a direct relationship between the higher score of EDII‐LIS and the increased likelihood of abdominal obesity (OR_WC_: 15.0, 95% CI: 12.3–18.3, *p* trend <.001, OR_WHR_: 2.68, 95% CI: 2.18–3.29, *p* trend <.001). Greater adherence to the EDII score was associated with a higher odds of general obesity, but not abdominal obesity. Also, individuals with a higher score of LIS and EDII‐LIS are more prone to abdominal obesity.

## Graphical abstract

Greater adherence to the empirical dietary inflammatory index (EDII) score was associated with a higher odds of general obesity. Individuals with a higher score of lifestyle inflammation score (LIS) and EDII‐LIS are more prone to abdominal obesity. Association between EDII and LIS with obesity measures in Iranian adults. Odds ratios (OR) and 95% confidence intervals (CI) are presented. Higher EDII scores were associated with increased odds of general obesity (OR: 1.21, 95% CI: 1.04–1.41). Higher LIS was associated with greater odds of abdominal obesity based on waist circumference (WC) (OR: 37.0, 95% CI: 28.8–47.5) and waist‐to‐hip ratio (WHR) (OR: 3.30, 95% CI: 2.65–4.11). Greater EDII‐LIS scores were also directly associated with a higher likelihood of abdominal obesity by WC and WHR criteria.

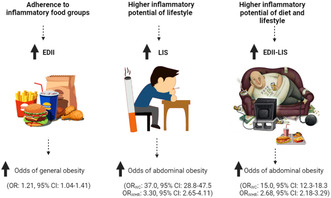

## 1 INTRODUCTION

Obesity is characterized by excessive secretion of pro‐inflammatory cytokines by adipose tissue, leading to a chronic low‐grade state of inflammation (Kim & Nam, 2020). Obesity can lead to the development of chronic diseases, including type 2 diabetes, hyperlipidemia, hypertension, coronary heart disease, and some cancers (Pi‐Sunyer, 2009). The prevalence of obesity has been progressively worsening over the past 50 years, affecting both developed and developing countries (Bhurosy & Jeewon, 2014). For example, in Middle Eastern nations, the prevalence of overweight and obesity is 54.2% in women and 31.4% in men, as reported by World Health Organization (WHO) (Khatib, 2004) (“Epidemiological update on prevalence and incidence of overweight and obesity in adults in the south‐east of the Islamic Republic of Iran: findings from KERCADRS”). Also, obesity prevalence in Iranian adults was estimated at 13% in 2019, which is higher than the average global rate (Pourfarzi et al., 2021). In recent years, major changes in lifestyle behaviors in the Iranian population have led to a high prevalence of obesity (Kelishadi et al., 2008).

Obesity has multiple contributing factors (Ayatollahi & Ghoreshizadeh, 2010), with lifestyle components, such as diet, smoking, and physical activity playing a significant role (Ward et al., 2015; Wing et al., 2001). Recent research shows that unhealthy eating habits, physical inactivity, and smoking may all cause inflammatory processes in the body, increasing the risk of chronic illnesses like obesity (Al‐Jawaldeh & Abbass, 2022). To assess the impact of diet and lifestyle on body inflammation, two new indices have been introduced: the empirical dietary inflammatory index (EDII) and the lifestyle inflammation score (LIS). The EDII measures the overall inflammatory potential of a diet based on food groups (Tabung et al., 2016), while the LIS assesses the influence of lifestyle components including BMI, physical activity, alcohol consumption, and smoking on inflammation (Byrd et al., 2019). According to recent research, higher EDII and LIS are associated with an increased risk of chronic diseases such as metabolic syndrome (MetS) (Farhadnejad et al., 2021), cancer (Byrd et al., 2020), and cardiovascular disease (CVD) risk factors (Li et al., 2021). However, to our knowledge, the association between EDII and LIS with obesity has not been studied; therefore, we aimed to examine this association among a large sample of Iranian adults.

## 2 MATERIALS AND METHODS

### 2.1 Study population

This cross‐sectional study was carried out using data from a prospective cohort study called “Yazd Health Study (YaHS)” (www.yahs‐ziba.com), which has been conducted on 10,000 participants aged 20–70 years since 2014. Detailed information about the design, data collection, sampling method, and baseline population of the YaHS study was published elsewhere (Mirzaei et al., 2017). In the present study, of 8965 available cases, subjects with chronic diseases including CVD, and different cancers (*n* = 855), pregnant women (*n* = 116), individuals with calorie intake less than 500 kcal/day or higher than 5000 kcal/day (*n* = 1572), and with missing data for BMI and WC (*n* = 89), were excluded. Finally, to investigate the association between EDII and obesity 6522 individuals were included in the analysis. In addition, participants with missing data on smoking (*n* = 182) and physical activity (*n* = 1000) were removed and 5374 participants were entered into the analysis on the association between LIS and obesity. Also, for the combinational index of EDII and LIS (EDII‐LIS), 5374 people remained to enter the final analysis. The methodology of the current study was approved by the Ethics Committee of Shahid Sadoughi University of Medical Sciences (No. 17/1/73941), and also by the Ethics Committee of Iran University of Medical Sciences (Ethical code: IR.IUMS.REC.1400.122).

### 2.2 Data measurement

Demographic information was collected using a pre‐tested questionnaire that measured data on age, sex, menopausal status, marital status, education level, family size, house ownership, employment status, medical history, and smoking habits.

### 2.3 Dietary assessment

Dietary intake was evaluated through the semi‐quantitative FFQ by trained interviewers. The original semi‐quantitative FFQ, which was previously validated for the Iranian population, has 168 items, but 10 more questions on the consumption of Yazd‐specific frequently consumed food items were added, which made a total of 178 food items (Zimorovat et al., 2022). Participants were asked to report the amount and frequency of consuming each food item each month, week, or day during the past year. Finally, by using household measures of consumed foods (Ghafarpour et al., 1999), we converted food items into grams per day.

### 2.4 Anthropometric and physical activity measurements

The participant's body weight was measured using a BF510 digital scale with an accuracy of 0.1 kg (Omron Inc.) in a standing position with light clothing. Anthropometric measurements were done before the interview, after completing one‐third of the questionnaire, and after completing two‐thirds of the questions consequently. Also, Participants' height was measured in a standing position using a tape measure on a straight wall to the nearest centimeter barefoot while their heads, shoulder blades, buttocks, and heels were rested against the wall. Then, BMI was calculated as weight (kg) divided by height in square meters (m^2^). While participants were in the standing position, waist circumference (WC) was measured to the nearest 0.5 cm using non‐stretch tape that was placed midway between the iliac crest and lowest rib (Edwards et al., 2008). Hip circumference was also measured over the largest part of the buttocks, with an accuracy of 0.5 cm. The waist‐to‐hip ratio (WHR) was calculated by dividing WC by hip circumference in centimeters.

The physical activity levels of the participants were assessed through the use of the Persian version of the International Physical Activity Questionnaire‐short form (IPAQ‐SF), which has been validated by Moghaddam et al. (2012). The data obtained were categorized into low, medium, or high levels of physical activity based on the guidelines in the IPAQ‐SF (Craig et al., 2003).

### 2.5 EDII and LIS calculation

The EDII score was calculated based on its development study conducted by Tabung et al. (2016). EDII has 18 components originally, but due to cultural constraints and lack of data on low‐energy drinks, wine, and alcohol, we calculated the overall score based on 15 food parameters including tea, coffee, dark yellow vegetables, leafy green vegetables, snacks, fruit juice, pizza, processed meat, red meat, organ meat, other fish (canned tuna or fish), other vegetables (vegetables other than leafy green vegetables and dark yellow vegetables), refined grains, high‐energy beverages, and tomatoes.

The mean daily intake of each food group (serving size) was calculated for each participant, then their intakes of each mentioned food group were multiplied by the proposed regression coefficients, and then all the computed values for each food group were summed and the final EDII score was calculated (Tabung et al., 2016). Finally, the EDII score is divided by 1000 to decrease the degree of the score and its simplicity of interpretation. A higher EDII score indicates a more pro‐inflammatory diet and vice versa. For data analysis and population stratification, the EDII was computed for each participant per 1000 kcal of energy intake.

The LIS score developed by Byrd et al. (2019) includes four components: smoking status, physical activity, alcohol intake, and BMI. Due to religious restrictions in the Iranian population, alcohol is not consumed, or its consumption is not reported, so we ignored alcohol intake to calculate LIS. We first created a dummy variable from each component and then multiplied the components by the regression coefficients, as follows: Physical activity was categorized into tertiles, and participants in the first, second, and third tertiles gave 0.0, −0.18, and −0.41, respectively. Participants were also categorized based on their BMI into average weight (BMI < 25), overweight (25 ≤ BMI < 30), and obese (BMI ≥ 30) with scores of 0.0, 0.89, and 1.57, respectively. Participants who identified as smokers were given a score of 0.50, while non‐smokers received a score of 0.0. Finally, the LIS score was calculated by summing up all the weighted values.

To create EDII‐LIS index, the scores of each of the mentioned indices were converted into *Z*‐score to equalize their weight, then EDII‐LIS index was created from the sum of the *Z*‐score of EDII and *Z*‐score of LIS indices.

### 2.6 Outcome definition

General obesity was defined based on WHO criteria as BMI ≥30 kg/m^2^ (WHO Consultation on Obesity, & World Health Organization, 2000). Also, for the definition of abdominal obesity, we used two anthropometric variables. Abdominal obesity_WC_ was defined using the cut‐off of WC ≥89 cm for men and ≥91 cm for women (Delavari et al., 2009) and abdominal obesity_WHR_ was defined using the cutoff of WHR ≥1 for men and ≥0.8 for women (WHO Consultation on Obesity, & World Health Organization, 2000).

### 2.7 Statistical analysis

Data analyses were conducted using SPSS software version 20. The normality of the data was assessed using the Kolmogorov–Smirnov test and histogram charts. The EDII and LIS scores were categorized into tertiles, and then the characteristics of the participants were represented accordingly. Data are presented as the mean and standard variation (mean ± SD) or the median (interquartile range) for continuous variables and percentages for categorical variables across tertiles of EDII and LIS. To test the trend of qualitative and quantitative variables across tertiles of EDII and LIS (as the median value in each tertile), Chi‐square and linear regression were used, respectively. Since BMI is a component of the LIS, it is methodologically impossible to assess the association between LIS and EDII‐LIS scores with general obesity, as general obesity is defined based on BMI. Therefore, we only evaluated the association between LIS and EDII‐LIS with abdominal obesity. We applied binary logistic regression analysis to estimate the odds ratios (ORs) and 95% confidence intervals (CIs) of general and abdominal obesity across tertiles of the scores in the crude and multivariable‐adjusted models. To determine the association between EDII and obesity indices, in addition to the crude model, age and sex were adjusted in model 1. Model 2 was further adjusted for smoking, physical activity, diabetes, thyroid disorders, marital status, socioeconomic status, menopausal status, and dietary intake of energy. LIS and EDII‐LIS scores were also adjusted for potential confounders including age, sex, diabetes, thyroid disorders, marital status, socioeconomic status, menopausal status, and dietary intake of energy. *p*‐value < .05 was considered statistically significant.

## 3 RESULTS

General characteristics and dietary intakes of participants across tertiles of EDII are presented in Table 1. Compared with those in the lowest tertiles of EDII, significant differences were observed for the variables of age, level of education, and menopausal status of the participants. No significant difference was detected in the anthropometric data of the participants according to the tertiles of EDII. It was found that all dietary intakes except for fiber and pizza had significant differences between participants according to the tertiles of EDII score. Individuals in the highest tertile of the EDII score had lower intakes of carbohydrates, polyunsaturated fatty acids (PUFA), tea, coffee, leafy green and dark yellow vegetables, snacks, and fruit juice than those in the lowest tertile. However, dietary intakes of energy, protein, fat, processed meats, red and organ meats, other fish, other vegetables, refined grains, and high‐energy beverages significantly increased across EDII score tertiles.

**TABLE 1:** Study population characteristics based on the tertiles of EDII score (per 1000 kcal) among the Yazd Health Study.

Demographic data	T1 (*n* = 2174)	T2 (*n* = 2174)	T3 (*n* = 2174)	*p* for trend^a^
Age (year)				**.041**
20–29 years (%)	19.1	20.7	22.7	
30–39 years (%)	20.8	20.2	21.2	
40–49 years (%)	22.0	22.1	21.7	
50–59 years (%)	20.3	19.0	19.3	
60–69 years (%)	17.8	18.0	14.9	
Male (%)	50.4	50.0	50.0	.953
Body mass index (kg/m^2^)	26.8 (5.18)	27.1 (5.27)	27.0 (5.08)	.530
Waist (cm)	93.6 (13.2)	93.9 (13.0)	93.3 (13.0)	.522
WHR	0.92 (0.10)	0.92 (0.09)	0.92 (0.10)	.804
Physical activity (MET/min/week)	1004 (890)	1034 (913)	1033 (888)	.081
Smoking (yes, %)	11.7	9.5	10.7	.059
Menopausal status (yes, %)	18.9	19.4	16.2	**.013**
Marital status (married, %)	85.4	84.2	84.0	.399
Education level (diploma and higher, %)	43.8	47.6	49.4	**.001**
Family size (>4 member, %)	24.8	27.2	27.4	.099
House acquisition (yes, %)	78.9	79.3	78.6	.821
Occupation status (employed, %)	80.9	81.3	81.0	.945
Socio economic status (%)				.807
Low (%)	33.3	32.3	33.3	
Middle (%)	46.3	46.2	45.0	
High (%)	20.5	21.4	21.7	
Diabetes (%)	12.8	13.4	12.3	.563
Thyroid disorders (%)	7.4	8.2	8.0	.582
Dietary intake				
Energy intake (kcal/day)	2511 (939)	2475 (929)	2642 (1040)	**<.001**
Carbohydrate (% of energy)	53.6 (8.21)	53.1 (7.41)	52.6 (7.82)	**<.001**
Protein (% of energy)	15.3 (3.90)	15.8 (3.59)	15.6 (4.15)	**.010**
Fat (% of energy)	31.0 (7.50)	30.9 (6.51)	31.6 (6.58)	**.002**
Polyunsaturated fatty acids (% of energy)	8.28 (5.36)	8.06 (4.70)	7.85 (3.79)	**.003**
Fiber (g/1000 kcal)	9.20 (4.51)	9.06 (3.89)	9.29 (4.12)	.415
Dietary components of EDII				
Positive associations				
Processed meat (serving/day)	0.25 (0.25–0.61)	0.25 (0.25–0.67)	0.25 (0.25–0.75)	**<.001**
Red meat (serving/day)	0.24 (0.12–0.42)	0.27 (0.16–0.45)	0.27 (0.14–0.49)	**<.001**
Organ meat (serving/day)	0.03 (0.02–0.07)	0.03 (0.02–0.08)	0.04 (0.02–0.14)	**<.001**
Other fish (serving/day)	0.05 (0.03–0.12)	0.08 (0.04–0.16)	0.08 (0.03–0.19)	**<.001**
Other vegetables (serving/day)	0.93 (0.53–1.39)	1.27 (0.85–1.95)	2.09 (1.12–3.04)	**<.001**
Refined grains (serving/day)	1.77 (1.29–2.41)	2.11 (1.54–3.17)	2.82 (1.68–6.41)	**<.001**
High energy beverages (serving/day)	0.06 (0.01–0.14)	0.06 (0.01–0.14)	0.06 (0.01–0.28)	**<.001**
Tomatoes (serving/day)	0.16 (0.08–0.48)	0.48 (0.16–0.48)	0.48 (0.16–1.13)	**<.001**
Inverse associations				
Tea (serving/day)	1.50 (0.95–2.87)	1.43 (0.47–2.87)	0.95 (0.13–1.91)	**<.001**
Coffee (serving/day)	0.008 (0.008–0.32)	0.008 (0.008–0.32)	0.008 (0.008–0.32)	**.049**
Dark yellow vegetables (serving/day)	0.13 (0.05–0.39)	0.12 (0.06–0.26)	0.11 (0.04–0.25)	**<.001**
Leafy green vegetables (serving/day)	0.26 (0.12–0.57)	0.21 (0.11–0.43)	0.16 (0.08–0.38)	**.049**
Snacks (serving/day)	0.51 (0.18–1.76)	0.43 (0.18–0.93)	0.34 (0.18–0.81)	**<.001**
Fruit juice (serving/day)	0.15 (0.05–0.47)	0.10 (0.05–0.32)	0.10 (0.05–0.32)	**.049**
Pizza (serving/day)	0.04 (0.04–0.16)	0.04 (0.04–0.16)	0.04 (0.04–0.16)	.232

*Note*: Data are presented as the mean ± SD or as the median (interquartile range) for continuous variables and as percentages for categorical variables.

Abbreviation: WHR, waist hip ratio.

^a^ Chi‐square and linear regression were used to test the trend of qualitative and quantitative variables across tertiles of EDII score.

We also presented the baseline characteristics and dietary intakes of subjects according to the tertiles of LIS in Table 2. Participants in the highest LIS tertiles were more likely to be female, older, married, in a postmenopausal state, smoked more, had more diabetes and thyroid disorders, and had lower physical activity, academic education levels, and socioeconomic status than those in the lowest LIS tertiles of LIS. Additionally, BMI, WC, and WHR were increased significantly across LIS tertiles. Furthermore, participants in the highest tertile of LIS had a higher intake of fiber, while the intake of other nutrients did not significantly differ across LIS tertiles.

**TABLE 2:** Study population characteristics based on the tertiles of LIS among the Yazd Health Study.

Demographic data	T1 (*n* = 1715)	T2 (*n* = 2133)	T3 (*n* = 1526)	*p* for trend^a^
Age (year)				**<.001**
20–29 years (%)	39.4	14.9	8.7	
30–39 years (%)	22.6	23.6	17.8	
40–49 years (%)	15.5	22.9	30.4	
50–59 years (%)	10.7	21.0	26.4	
60–69 years (%)	11.9	17.6	16.8	
Male (%)	52.3	54.2	43.1	**<.001**
Body mass index (kg/m^2^)	21.9 (2.23)	26.6 (2.42)	32.8 (4.20)	**<.001**
Waist (cm)	82.9 (9.38)	93.9 (9.54)	104 (10.4)	**<.001**
WHR	0.89 (0.12)	0.93 (0.09)	0.94 (0.09)	**<.001**
Physical activity (MET/min/week)	1119 (955)	1030 (896)	906 (817)	**<.001**
Smoking (yes, %)	0	12.0	20.1	**<.001**
Menopausal status (yes, %)	8.9	17.1	26.4	**<.001**
Marital status (married, %)	76.0	87.0	90.6	**<.001**
Education level (diploma and higher, %)	61.1	46.7	37.2	**<.001**
Family size (>4 member, %)	27.4	26.3	27.1	.731
House acquisition (yes, %)	75.2	79.2	83.2	**<.001**
Occupation status (employed, %)	79.9	80.3	82.9	.062
Socioeconomic status (%)				**<.001**
Low (%)	29.8	34.0	33.7	
Middle (%)	44.7	44.2	47.9	
High (%)	25.5	21.8	18.4	
Diabetes (%)	6.8	13.6	16.7	**<.001**
Thyroid disorders (%)	6.5	7.5	10.2	**<.001**
Dietary intake				
Energy intake (kcal/day)	2562 (953)	2541 (966)	2551 (975)	.686
Carbohydrate (% of energy)	53.03 (7.87)	52.9 (7.86)	53.2 (7.93)	.635
Protein (% of energy)	15.5 (3.86)	15.6 (3.98)	15.6 (3.89)	.347
Fat (% of energy)	31.3 (6.81)	31.3 (6.83)	31.1 (6.97)	.279
Polyunsaturated fatty acids (% of energy)	8.12 (4.98)	8.08 (4.64)	7.92 (3.89)	.222
Fiber (g/1000 kcal)	8.98 (3.33)	9.17 (4.67)	9.31 (4.29)	**.022**

*Note*: Data are presented as the mean ± SD or as the median (interquartile range) for continuous variables and as percentages for categorical variables.

Abbreviation: WHR, waist hip ratio.

^a^ Chi‐square and linear regression were used to test the trend of qualitative and quantitative variables across tertiles of LIS.

The association between EDII and general obesity, as well as two definitions of abdominal obesity based on WC and WHR, is presented in Table 3. Compared with participants in the first tertile of EDII, based on the crude model, adjusted models 1 and 2, participants in the third tertile had higher odds of general obesity (OR: 1.21, 95% CI: 1.04–1.41, *p* trend: .016). However, there was no association between EDII and abdominal obesity either in the crude model or in models 1 and 2 (OR_WC_: 1.10, 95% CI: 0.92–1.31, *p* trend: .459 and OR_WHR_: 1.06, 95% CI: 0.92–1.22, *p* trend: .212).

**TABLE 3:** The association between the empirically dietary inflammatory index with general and abdominal obesity^a^.

	Tertiles of EDII (per 1000 kcal)			*p* for trend
	T1	T2	T3	
General obesity				
Median score	0.07	0.16	0.29	
Case/Total	518/2174	557/2174	565/2174	
Crude model	1.00 (Ref)	1.10 (0.94–1.27)	1.16 (1.00–1.34)	**.044**
Model 1^b^	1.00 (Ref)	1.11 (0.95–1.29)	1.21 (1.04–1.41)	**.013**
Model 2^c^	1.00 (Ref)	1.11 (0.96–1.30)	1.21 (1.04–1.41)	**.016**
Abdominal obesity (WC)				
Median score	0.07	0.16	0.29	
Case/Total	1365/2174	1392/2174	1348/2174	
Crude model	1.00 (Ref)	0.98 (0.86–1.11)	1.05 (0.92–1.19)	.903
Model 1^b^	1.00 (Ref)	0.97 (0.81–1.15)	1.11 (0.93–1.32)	.367
Model 2^c^	1.00 (Ref)	0.97 (0.81–1.15)	1.10 (0.92–1.31)	.459
Abdominal obesity (WHR)				
Median score	0.07	0.16	0.29	
Case/Total	1150/2174	1142/2174	1151/2174	
Crude model	1.00 (Ref)	1.05 (0.92–1.20)	0.99 (0.87–1.13)	.385
Model 1^b^	1.00 (Ref)	1.09 (0.94–1.25)	1.07 (0.93–1.23)	.199
Model 2^c^	1.00 (Ref)	1.08 (0.94–1.25)	1.06 (0.92–1.22)	.212

^a^ Logistic regression models were used to obtain OR with 95% CIs in obesity outcomes across tertiles (T) of EDII.

^b^ Model 1: adjusted for age and sex.

^c^ Model 2: adjusted for model 1 and smoking, physical activity, diabetes, thyroid disorders, marital status, socioeconomic status, menopausal status, and dietary intake of energy.

Table 4 shows the results on the association between LIS and the odds of abdominal obesity. In the crude model, there were increased odds of abdominal obesity for subjects at the highest compared to the lowest tertile of the LIS (OR_WC_: 38.4, 95% CI: 30.3–48.8, *p* for trend <.001 and OR_WHR_: 3.01, 95% CI: 2.59–3.50, *p* for trend <.001). The same was observed in the age and sex‐adjusted model. Also after further adjustment for diabetes, thyroid disorders, marital status, socioeconomic status, menopausal status, and dietary intake of energy in the multivariable‐adjusted model, the direct association of LIS and abdominal obesity remained significant (OR_WC_: 37.0; 95% CI: 28.8–47.5, *p* for trend <.001 and OR_WHR_: 3.30; 95% CI: 2.65–4.11, *p* for trend <.001).

**TABLE 4:** The association between the lifestyle inflammatory score with abdominal obesity^a^.

	Tertiles of LIS			*p* for trend
	T1	T2	T3	
Abdominal obesity (WC)				
Median score	−0.18	0.71	1.39	
Case/Total	447/1715	1501/2133	1416/1526	
Crude model	1.00 (Ref)	6.64 (5.72–7.70)	38.4 (30.3–48.8)	**<.001**
Model 1^b^	1.00 (Ref)	6.06 (5.18–7.09)	37.3 (29.1–47.7)	**<.001**
Model 2^c^	1.00 (Ref)	6.03 (5.15–7.07)	37.0 (28.8–47.5)	**<.001**
Abdominal obesity (WHR)				
Median score	−0.18	0.71	1.39	
Case/Total	706/1715	1112/2133	1032/1526	
Crude model	1.00 (Ref)	1.52 (1.32–1.73)	3.01 (2.59–3.50)	**<.001**
Model 1^b^	1.00 (Ref)	1.84 (1.52–2.23)	3.50 (2.82–4.35)	**<.001**
Model 2^c^	1.00 (Ref)	1.77 (1.46–2.15)	3.30 (2.65–4.11)	**<.001**

^a^ Logistic regression models were used to obtain OR with 95% CIs in abdominal obesity across tertiles (T) of LIS.

^b^ Model 1: adjusted for age and sex.

^c^ Model 2: adjusted for model 1 and diabetes, thyroid disorders, marital status, socioeconomic status, menopausal status, and dietary intake of energy.

Table 5 presents the results of the combined role of the inflammatory potential of diet and lifestyle, as determined by EDII‐LIS, in predicting the likelihood of abdominal obesity. In the crude model, the higher score of EDII‐LIS was associated with increased odds of abdominal obesity (OR_WC_: 13.1, 95% CI: 11.0–15.7, *p* for trend <.001 and OR_WHR_: 2.81, 95% CI: 2.44–3.24, *p* for trend <.001). This positive association remained significant in adjusted models for potential confounders and in the final adjusted model the OR (95% CI) of abdominal obesity_WC_ and abdominal obesity_WHR_ were 15.0 (12.3–18.3), *p* for trend <.001 and 2.68 (2.18–3.29), *p* for trend <.001, respectively.

**TABLE 5:** The association between the dietary and lifestyle inflammatory score (EDII combination with LIS) with abdominal obesity^a^.

	Tertiles of EDII combination with LIS			*p* for trend
	T1	T2	T3	
Abdominal obesity (WC)				
Median score	−1.36	−0.05	1.20	
Case/Total	593/1792	1221/1790	1550/1792	
Crude model	1.00 (Ref)	4.31 (3.73–5.00)	13.1 (11.0–15.7)	**<.001**
Model 1^b^	1.00 (Ref)	4.35 (3.72–5.09)	13.6 (11.3–16.4)	**<.001**
Model 2^c^	1.00 (Ref)	4.49 (3.83–5.28)	15.0 (12.3–18.3)	**<.001**
Abdominal obesity (WHR)				
Median score	−1.36	−0.05	1.20	
Case/Total	717/1792	974/1790	1159/1792	
Crude model	1.00 (Ref)	1.18 (1.57–2.08)	2.81 (2.44–3.24)	**<.001**
Model 1^b^	1.00 (Ref)	1.87 (1.55–2.27)	2.81 (2.30–3.42)	**<.001**
Model 2^c^	1.00 (Ref)	1.82 (1.50–2.21)	2.68 (2.18–3.29)	**<.001**

^a^ Logistic regression models were used to obtain OR with 95% CIs in abdominal obesity across tertiles (T) of EDII‐LIS score.

^b^ Model 1: adjusted for age and sex.

^c^ Model 2: adjusted for model 1 and diabetes, thyroid disorders, marital status, socioeconomic status, menopausal status, and dietary intake of energy.

## 4 DISCUSSION

In this cross‐sectional study, EDII and LIS were used to assess the inflammatory potential of diet and lifestyle on a large sample of non‐obese adults in Yazd regarding obesity outcomes. The results revealed a significant association between higher EDII scores and general obesity, but not with abdominal obesity. Additionally, higher scores of LIS and EDII‐LIS were found to significantly increase the odds of abdominal obesity. To the best of our knowledge, this is the first study that has evaluated the association of EDII and LIS with obesity.

Evidence suggests inflammation might contribute to the onset of obesity and the relationship between obesity and inflammation is bidirectional (Sears & Ricordi, 2011). Individual levels of systemic inflammation are influenced by several environmental factors, such as diet, exercise, smoking, and alcohol consumption (Guarner & Rubio‐Ruiz, 2015). In this study, it was found that participants with pro‐inflammatory diets and lifestyles were more likely to develop obesity. Although to date, no study has examined the relationship between EDII and LIS in obese patients; EDII is associated with increased odds of obesity in our study in a similar way to previous studies assessing the role of DII in obesity development. For instance, a study by Ramallal et al. (2017) in the SUN cohort found that higher DII scores were associated with an increased risk of obesity. Similarly, the CUME research found that among Brazilian adults, the highest pro‐inflammatory quartile of DII was associated with a greater prevalence of obesity (Oliveira et al., 2020). Besides, results of a recent meta‐analysis of four studies assessing the relationship between DII and obesity showed that a pro‐inflammatory diet is associated with a higher risk of obesity (pooled OR: 1.31; 95% CI: 1.14, 1.50) (Kord Varkaneh et al., 2018). Some cross‐sectional studies, however, have found no association between diet‐induced inflammation measured by DII or EDII and obesity (Haji‐Hosseini‐Gazestani et al., 2020; Mokhtary et al., 2020; San et al., 2018). The discrepancy between our results and those with no association may be caused by inconsistencies in study design, population, assessed food items, tools used to evaluate dietary intake, dietary indices (e.g., DII, which emphasize nutrients as opposed to food groups in the present study), and the impact of unmeasurable confounding factors.

The present study does not demonstrate a significant association between EDII and abdominal obesity. In line with our findings, some research also found no significant association between the DII score and WC (Neufcourt et al., 2015; Wirth et al., 2014). While in the previous cohort (Khan et al., 2020; Shakeri et al., 2019) and cross‐sectional studies (Ruiz‐Canela et al., 2015; Sokol et al., 2016) a significant positive association was found. This observation may be due to the higher age of the participants, which is associated with elevated inflammation (Hartanto et al., 2021), having a more pro‐inflammatory diet, and, as a result, higher scores of inflammatory indices in these studies.

Unlike the EDII, the LIS was associated with increased odds of abdominal obesity in the present study. In other words, the high inflammatory potential of lifestyle may increase obesity likelihood by influencing abdominal obesity indicators. It seems that a higher score of LIS is related to an increased risk of insulin resistance (IR) (Farhadnejad et al., 2022) which appears to be associated with increased abdominal fat (Frayn, 2000). Based on baseline findings, individuals in the third tertile of LIS were largely smokers and inactive. Each of these environmental variables can be just as important as the dietary pattern in increasing inflammatory states and predisposing individuals to chronic diseases such as obesity. Inactivity is linked to lifestyle‐related chronic diseases through low‐grade inflammation, as measured by increased levels of c‐reactive protein (CRP), interlukin‐6 (IL‐6), and tumor necrosis factor α (TNF‐α) (Fischer et al., 2007; Strohacker & McFarlin, 2010). Moderate to high physical activity, however, is protective against chronic inflammation by reducing lipid deposits and reducing body fat through a negative energy balance that induces anti‐inflammatory cytokines (DiMenna & Arad, 2018). Also, smoking has a detrimental impact on metabolism, β‐cells dysfunction, and IR, which are all linked to increased levels of inflammatory biomarkers and cytokines such as CRP (Stadler et al., 2014). It has previously been reported that current smokers tend to have a larger WC and a higher WHR than non‐smokers, suggesting that smoking may induce the accumulation of abdominal fat (Akbartabartoori et al., 2005). This claim can be supported by the results of abdominal obesity indices across LIS tertiles, which revealed that an unfavorable environmental factor such as low physical activity can worsen the inflammatory condition and predispose individuals to obesity. Therefore, the contributions of lifestyle‐related inflammatory determinants, such as physical activity and smoking, might have a stronger relationship with increased odds of abdominal obesity than EDII. As we know, there has been no study on LIS and obesity. So, we suggest conducting studies to assess the potential association between LIS and obesity.

Since the EDII only focuses on diet and the LIS only includes lifestyle and non‐dietary factors, we created the combined EDII‐LIS index, which is a more comprehensive index that includes both dietary and non‐dietary factors. Based on our results, a higher EDII‐LIS score has a significant positive association with abdominal obesity. In other words, those with a higher EDII‐LIS score tended to adhere to an unhealthy dietary pattern and unhealthy lifestyle that may contribute to a greater odds of abdominal obesity. The combined effect of dietary and lifestyle indices on chronic disease risk has been investigated in recent years. For example, the results of recent studies showed a direct association between the diet and lifestyle inflammation score (DLIS) and the risk of IR and metabolic syndrome (Dehghani Firouzabadi et al., 2021; Farhadnejad et al., 2022). There is a bidirectional relationship between obesity and IR (Xu et al., 2021) and the most severe form of IR is associated with fat distribution in the abdominal area (Cheng et al., 2017). Each of the components of this index is involved in increasing inflammatory cytokines that can contribute to IR and therefore the chance of abdominal obesity. Having a higher BMI is highly correlated with IR (Lim et al., 2015). Also, physical inactivity and smoking can determine an important part of the high inflammatory effect of an unhealthy lifestyle through increased inflammatory cytokines, decreased insulin sensitivity, and increased IR (Eaton & Eaton, 2017; van der Vaart et al., 2004). In addition, a dietary pattern with a higher score of EDII is characterized by higher consumption of pro‐inflammatory food groups such as red, processed, and organ meats, other fish, other vegetables, high‐energy beverages, tomatoes, and refined grains and lower consumption of tea, coffee, snacks, fruit juice, dark yellow and leafy green vegetables, which could be strongly correlated with increased inflammatory markers levels and IR (Tabung et al., 2016, 2017). It may be that inflammation and IR drive abdominal obesity, which explains the possible role of these indices in increasing the risk of abdominal obesity.

The present study has several strengths. To begin, it was conducted in a Middle Eastern country with a population‐based prospective design and a large sample size for both sexes, and adjusted for many plausible confounders. Another strength of this study is the use of a valid and reliable FFQ, which provided an accurate calculation of EDII, and a validated physical activity questionnaire to assess energy expenditure for physical activity. To our knowledge, this is also the first study to identify the association between EDII and LIS with obesity.

However, our study has some limitations. Since it was a cross‐sectional study, a causal relationship could not be investigated and prospective studies are needed to confirm these findings. Moreover, some items could not be included in the calculations of EDII and LIS, which may have affected the results. Furthermore, the lack of data on blood levels of inflammation markers limited our ability to conduct further investigation and thoroughly interpret the results.

## 5 CONCLUSION

In conclusion, an increased EDII score was significantly correlated with general obesity, but not with abdominal obesity. Also, our results suggest that a higher score of LIS and EDII‐LIS are associated with an increased odds of abdominal obesity in adults. It would be beneficial to carry out additional prospective studies with other designs in different populations to approve the present results.

## AUTHOR CONTRIBUTIONS

**Niloufar Saber:** Conceptualization (equal); formal analysis (equal); investigation (equal); writing – original draft (lead). **Mahdieh Hosseinzadeh:** Methodology (equal). **Sakineh Shab‐Bidar:** Formal analysis (equal). **Masoud Mirzaei:** Investigation (equal); methodology (equal). **Azadeh Najarzadeh:** Methodology (equal). **Seyedeh Tayebeh Rahideh:** Supervision (equal).

## CONFLICT OF INTEREST STATEMENT

The authors declare no conflicts of interest.

## ETHICS STATEMENT

All methods of this study were carried out under the Declaration of Helsinki's ethical principle for medical research involving human subjects. YaHS was approved by the Ethics Committee of Shahid Sadoughi University of Medical Sciences (No. 17/1/73941). Additionally, the Ethics Committee of Iran University of Medical Sciences approved the protocol of this study (IR.IUMS.REC.1400.122).

## CONSENT TO PARTICIPATE

All participants in the study provided written informed consent.

## ACCORDANCE STATEMENT

The authors declare that all methods were carried out in accordance with relevant guidelines and regulations.

## Data Availability

The datasets generated and analyzed during the current study are not publicly available due to our institute policies but are available from the corresponding author on reasonable request.
